# 

**Published:** 1845-10

**Authors:** 


### CONTENTS OF N° XL

OF THE

### British and Foreign Medical Review.

OCTOBER 1845.

---

### Part First.--Analytical and Critical Reviews.

---

PAGE

Art. I.—Anatomical and Pathological Observations. By John Goodsir, f.r.s.e., Demonstrator of Anatomy in the University of Edinburgh; and Harry D. S. Goodsir, m.w.s., Conservator of the Museum of the Royal College of Surgeons, Edinburgh . . . . . 289

Art. II.—Technik der Medicinischen Diagnostik. Von D. A. Siebert, &c. Erster Band . . . . . . . 308

The Art of Medical Diagnosis. By Dr. A. Siebert, &c.

Art. III.—A Treatise on Poisons in relation to Medical Jurisprudence, Physiology, and the Practice of Physic. By Robert Christison, m.d. f.r.s. e. &c. . 317

Art. IV. — Lehrbuch der Allgemeinen Krankheitslehre. Von Dr. Carl H. Schultz, &c. . . . . . . 339

Elements of General Pathology. By Dr. C. H. Schultz, Professor in the University of Berlin, &c. &c.

Art. V.—Report on Smallpox in Calcutta, 1833-4, 1837-8, 1843-4, and Vaccination in Bengal, from 1827 to 1844. By Duncan Stewart, m.d., Superintendent General of Vaccination. Published by Order of Government . 349

Art. VI.—Manuel Pratique des Maladies des Nouveaux-Nés, et des Enfants à la Mamelle, etc. Par E. Bouchut, d.m. etc. . . . 357

A Manual of the Diseases of Infants and Children at the Breast. By E. Bouchut, m.d.

Art. VII.—1. Traité Complet de l'Hypocondrie. Par J. L. Brachet, Professeur de Pathologie générale, President de la Société de Médecine de Lyon, &c. 364

A Complete Treatise on Hypochondria. Par J. L. Brachet, Professor of General Pathology, &c.

§

PAGE

2. Traité Pratique, Dogmatique, et Critique de l'Hypocondrie. Par C. F. Michéa, Docteur en Médecine de la Faculté de Paris. . . 346

A Practical, Dogmatic, and Critical Treatise on Hypochondria. By C. F. Michéa, M.D. of the Faculty of Paris.

Art. VIII.—1. Odontography; or a Treatise on the Comparative Anatomy of the Teeth; their Physiological Relations, Mode of Development, and Microscopic Structure, in the Vertebrate Animals. By Richard Owen, F.R.S. Correspondent of the Royal Academy of Sciences in Paris, Berlin, &c. &c. Hunterian Professor to the Royal College of Surgeons, London . 376

2. Report on the Microscopic Structure of Shells. Part I. By William B. Carpenter, M.D. F.R.S., Fullerian Professor of Physiology in the Royal Institution of Great Britain.

3. Observations on the Structure of the Shells of Molluscous and Conchiferous Animals. By John Scott Bowerbank, &c.

Art. IX.—Impversuche und natur-historisch pathologische Untersuchungen zur Erforschung der Helminthiasis als Contagium. Einer Inauguralschrift von P. F. H. Klencke. . . . . . . 406

Experiments in Inoculation, and Pathological Researches and Natural History to develop Helminthiasis as Contagion. An Inaugural Dissertation by P. F. H. Klencke.

Art. X.—Samuel Thomas von Soemmering, Lehre von den Eingeweiden und Sinnesorganen des menschlichen Körpers. Umgearbeitet und beendigt von G. Huschke . . . . . . 419

Samuel Thomas von Soemmering's History of the Viscera and the Organs of Sense in the Human Body. Edited and completed by G. Huschke.

Art. XI.—1. Contributions to a Knowledge of the Influence of Employments upon Health. 2. Further Contributions to a Knowledge of the Influence of Employments upon Health. 3. A Third Contribution to a Knowledge of the Influence of Employments upon Health. 4. Health of Towns Commission. Minutes of Evidence given by William Augustus Guy, M.B. on the Influence of Employments upon Health. By William AugustusGuy, M.B. Contab., Professor of Forensic Medicine, King's College, and Physician to King's College Hospital . . . . . . . 421

Art. XII.—Das Venensystem in seinen Krankhaften. Verhältnissen dargestellt von Dr. F. A. B Püchelt, &c. &c. Erster Theil. Venöser Zustand— Erhöhte Vernosität . . . . . 430

The Venous System in its Pathologieal Relations. By Dr. F. A. B. Püchelt, Privy Councillor, Ordinary Professor of Pathology in the University of Heidelberg, &c. &c. The First Part. The Venous State—Exalted Venosity.

Art. XIII.—Observations upon the Employment of Compression in Aneurism, with some Statistical Details. By O. B. Bellingham, M.D. F.R.C.S.I. Surgeon to St. Vincent's Hospital . . . . . 434

Art. XIV.—1. Guide du Médecin Praticien, ou Résumé géneral de Pathologie Interne et de Thérapeutique appliquées. Par F. L. J. Valleix, Médecin du Bureau central des Hôpitaux de Paris, etc. . . . 441

Guide of the Practical Physician, or General Summary of Internal Pathology and Therapeutics, clinically applied. By F. P. J. Valleix, Physician to the Central Bureau of Hospitals of Paris.

2. The Practice of Medicine; a Treatise on Special Pathology and Therapeutics. By R. Dunglison, M.D., Professor of the Institutes of Medicine in Jefferson Medical College, Philadelphia, &c.

PAGE

ART. XV.—Excentricités Chirurgicales, ou Nouveaux Mémoires pour servir à la réforme et au perfectionnement de la Médecine Opératoire. Par MATHIAS MAYOR . . . . . . 461

Surgical Eccentricities, or New Memoirs for reforming and perfecting Operative Medicine. By MATHIAS MAYOR.

ART. XVI.—An Inquiry into the Physiological and Medicinal Properties of the Aconitum Napellus. By A. FLEMING, M.D. President of the Royal Medical Society of Edinburgh . . . . . 464

ART. XVII.—General Reports of the Royal Hospitals of Bridewell and Bethlem, for the Years ending 31st December 1843 and 1844. Printed for the use of the Governors . . . . . . 471

ART. XVIII.—1. De Syphilide Kali hydriodico tractata, Dissertatio quam pro licentia summos in medicina honores rite capessendi publico eruditorum examini subjicit, auctor MARTINUS HASSING . . . 482

Dissertation on the Treatment of Syphilis by the Iodide of Potassium. By MARTIN HASSING.

2. Observations pratiques sur le traitement des Maladies Syphilitiques par l'Iodine de Potassium. Par le Docteur L. P. A. GAUTHIER.

Practical Observations on the Treatment of Syphilitic Diseases by the Iodide of Potassium. By Dr. GAUTHIER.

ART. XIX.—1. Monograph of the Class Myriapoda, Order Chilopoda; with Observations upon the General Arrangement of the Articulata. By GEORGE NEWPORT, Esq. Fellow of the Royal College of Surgeons, President of the Entomological Society, &c. . . . . . 487

2. On the Structure, Relations, and Development of the Nervous and Circulatory Systems, and on the existence of a complete Circulation of Blood in Vessels, in Myriapoda, and Macrourous Arachnida. First Series. By GEORGE NEWPORT, Esq. F.R.C.S. &c.

3. On the Reproduction of Lost Parts in Myriapoda and Insecta. By GEORGE NEWPORT, F.R.C.S. &c. &c.

ART. XX.—Transactions of the Provincial Medical and Surgical Association, vol. XIII. 508

Dr. COWAN's Retrospective Address on Practical Medicine . . 509

Dr. BUDD's Retrospect of Anatomy and Physiology for the year 1843-44 . ib.

Dr. HOCKEN on the treatment of the inflammatory affections of the retina . 510

Mr. CROSSE on inversio uteri . . . . . 511

Mr. GILES on congenital malformation of the urinary organs . . ib.

ART. XXI.—On Cataract, and its appropriate Treatment by the Operation adapted to each Case. By CHARLES GARDINER GUTHRIE, Assistant Surgeon to the Royal Westminster Ophthalmic Hospital . . . . 512

### PART SECOND.—Bibliographical Notices.

PAGE

ART. I.—1. On Asthenopia, or Weaksightedness. By WM. MACKENZIE, M.D. Surgeon Oculist in Scotland in Ordinary to her Majesty, &c. . . 517

2. On the Vision of Objects on and in the Eye. By WM. MACKENZIE, M.D. &c. ib.

ART. II.—Human Health; or the Influence of Atmosphere and Locality; Change of Air and Climate; Seasons; Food; Clothing; Bathing and Mineral Springs; Exercise; Sleep; Corporeal and Intellectual Pursuits, &c. &c. on Healthy Man, constituting Elements of Hygiene. By ROBLEY DUNGLISON . 519

ART. III.—Mental Maladies; a Treatise on Insanity. By E. ESQUIROL. Translated from the French, with Additions, by E. K. HUNT, M.D. . 520

ART. IV.—Surgical and Practical Observations on the Diseases of the Human Foot, with Instructions for their Treatment. To which is added Advice on the Management of the Hand. By JOHN EISENBERG . . . 521

ART. V.—A Practical Treatise on the Special Diseases of the Skin. By C. M. GIBERT, Physician to the Hôspital St. Louis, &c. Translated from the French by EDGAR SHEPPARD . . . . . . ib.

ART. VI.—Remarks upon the Mortality of Exeter; together with Suggestions towards the Improvement of the Public Health. Being a Letter addressed to Henry Hooper, Esq. the right Worshipful the Mayor of Exeter. By THOMAS SHAPTER, M.D. &c. . . . . . . 523

### PART THIRD.—Original Reports and Memoirs.

Report on the Progress of Practical Medicine in the departments of Midwifery and the Diseases of Women and Children, during the years 1844-5. By CHARLES WEST, M.D. Member of the Royal College of Physicians; Physician to the Royal Infirmary for Children; Lecturer on Midwifery at the Middlesex Hospital; and Physician-accoucheur to the Finsbury Dispensary . . 525

INDEX, TITLE, &C.

